# Local Environment of Sc and Y Dopant Ions in Aluminum Nitride Thin Films

**DOI:** 10.1021/acsaelm.3c01390

**Published:** 2024-01-19

**Authors:** Asaf Cohen, Junying Li, Hagai Cohen, Ifat Kaplan-Ashiri, Sergey Khodorov, Ellen J. Wachtel, Igor Lubomirsky, Anatoly I. Frenkel, David Ehre

**Affiliations:** † Department of Molecular Chemistry and Materials Science, Weizmann Institute of Science, Rehovot 7610001, Israel; ‡ Department of Chemical Research Support, Weizmann Institute of Science, Rehovot 7610001, Israel; § Department of Materials Science and Chemical Engineering, Stony Brook University, Stony Brook, New York 11794, United States

**Keywords:** aluminum scandium nitride, aluminum yttrium nitride, sputtering, seeding layer, texture, piezoelectric

## Abstract

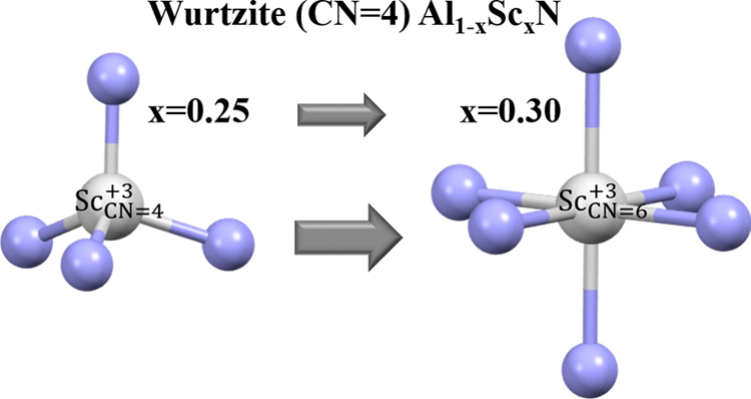

The local environments of Sc and Y in predominantly ⟨002⟩ textured, Al_1–*x*_Do_*x*_N (Do = Sc, *x* = 0.25, 0.30 or Y, *x* = 0.25) sputtered thin films with wurtzite symmetry were investigated using X-ray absorption (XAS) and photoelectron (XPS) spectroscopies. We present evidence from the X-ray absorption fine structure (XAFS) spectra that, when *x* = 0.25, both Sc^3+^ and Y^3+^ ions are able to substitute for Al^3+^, thereby acquiring four tetrahedrally coordinated nitrogen ligands, i.e., coordination number (CN) of 4. On this basis, the crystal radius of the dopant species in the wurtzite lattice, not available heretofore, could be calculated. By modeling the scandium local environment, extended XAFS (EXAFS) analysis suggests that when *x* increases from 0.25 to 0.30, CN for a fraction of the Sc ions increases from 4 to 6, signaling octahedral coordination. This change occurs at a dopant concentration significantly lower than the reported maximum concentration of Sc (42 mol % Sc) in wurtzite (Al, Sc)N. XPS spectra provide support for our observation that the local environment of Sc in (Al, Sc)N may include more than one type of coordination.

## Introduction

During the search for lead-free, Si-microfabrication compatible piezoelectric materials, thin films of doped aluminum nitride (AlN) have stimulated considerable interest. As actuators,^1^ energy harvesters^2^ and as components of microelectromechanical-systems (MEMS),^3^ they show promising results. Among the reasons for applications of AlN in MEMS devices are its high-temperature stability, high Curie temperature (*T*_c_ = 1423 K), and low relative dielectric permittivity (∼10).^3^ However, the piezoelectric constants of AlN thin films are relatively low compared to those of other piezoelectric materials, and metal doping has been shown to be beneficial in this regard. Among the dopants used to increase the material piezoelectric coefficients are codopants magnesium/niobium,^4^ trivalent scandium^5,6^ and the less costly, trivalent yttrium.^3^ Notably, *c*-axis-tilted (Al, Y)N thin films display high shear electromechanical coupling constants, which makes them very promising, high-performance materials for surface acoustic wave (SAW) and bulk acoustic wave (BAW) detection.^7^

However, preparation of (Al, Sc)N films is challenging because ScN and YN (both rock-salt-type (*Fm*3̅*m*) symmetry, space group #225) and AlN (wurtzite-type *P*6_3_*m*, polar space group #176) are totally immiscible.^8^ As a result, substitutional solid solutions of AlN with ScN or YN are thermodynamically unstable under ambient conditions and undergo phase segregation. This instability has been attributed, at least in part, to the considerable disparity in crystal radius between aluminum (67.5 pm) and scandium or yttrium (88.5 or 104 pm, respectively), for coordination number (CN) 6.^9^ Lowering CN to 4, would be expected to further reduce the crystal radius for all three elements, although a value has been determined only for Al (53 pm). Consequently, successful deposition of the metastable phases of AlDoN (Do = Sc or Y), with controlled *c*-axis orientation, a desirable property for piezoelectric applications, presents a significant challenge. Reactive sputtering allows synthesizing solid solutions away from thermodynamic equilibrium, so even metastable phases can be obtained, in spite of the internal driving force toward phase separation into domains with different crystal structures. Additional deposition parameters which must be optimized include: temperature,^6^ deposition pressure,^10^ seed-layer epitaxy,^11^ and substrate surface roughness.^12^

While incorporation of these dopants into the AlN wurtzite phase has been demonstrated by X-ray diffraction (XRD), i.e., progressive increase in the lattice parameters and in the piezoelectric response,^6,13,14^ little is known concerning the local environment of the dopant. Akiyama et al. demonstrated incorporation of the Sc^3+^ ion into the wurtzite phase at mole fraction ≤42%, beyond which phase separation occurs, even upon sputtering.^6,13^ This study also found that, as the concentration of Sc increased to 27 mol %, the 002 diffraction peak of the wurtzite phase moved to lower diffraction angles, i.e., the *c*-axis periodicity increased. However, beyond 27 mol %, the 002-peak moved to higher diffraction angles. The significance of the concentration at which this reversal occurred has not been addressed. The authors suggested that development of local stress/strain during the deposition process might have contributed to phase segregation of ScN. Incorporation of Y into the AlN wurtzite phase is even more restrictive.^15^

The present work investigates the effective size and local environment of trivalent Sc and Y ions within the host AlN wurtzite lattice. Extended X-ray absorption fine structure (EXAFS) spectroscopy and X-ray photoelectron spectroscopy (XPS) measurements provide evidence that, for both Sc and Y, the majority of the dopant ions, upon substitution for an aluminum ion, acquire four N atoms as near neighbors. For the case of Sc, we also observed that, at 30 mol % doping, a minor fraction of the ions are coordinated by 6, rather than 4, nitrogen ions. We were able to discount the possibility of Sc^3+^ coordinated by 5 nitrogen ions. There are no literature reports of binary compounds of 5-coordinated Sc. A successful attempt to stabilize 5-coordinated, trivalent Sc, involving multicomponent (including P, N, Cl, O, and Si) organometallic synthesis, has in fact been described.^16^ However, that possibility is not relevant to the structures investigated here. Consequently, we proposed either a homogeneous model (all Sc atoms are in a 4-coordinated state) or a heterogeneous model (Sc atoms partition between 4 and 6-coordinated states). The second model is the more probable for Al_0.70_Sc_0.30_N.

These findings support the concept that the phase separation observed at 42 mol % Sc must, in fact, be the culmination of a gradual process. Additionally, by calculating the number of nearest neighbors and bond lengths obtained from XAS measurements, we are able to report estimates of the effective size of Sc_CN=4_^3+^ and Y_CN=4_^3+^ in the wurtzite lattice.

## Experimental Section

### Materials

N_2_, argon, and O_2_ sputtering gases (Gas Technologies, Israel, 99.9999 purity) were used. Hydrofluoric acid (HF), organic solvents, acetone, and isopropyl alcohol (IPA) were semiconductor CMOS grade (Sigma-Aldrich). Scandium nitride (ScN) powder (99.9% purity, Sigma-Aldrich) was used for EXAFS measurements.

### Deposition of Al_1–*x*_Do*_x_*N (Do = Sc, *x* = 0.25, 0.30 or Y, *x* = 0.25) Thin Films

(Al_1–*x*_Do_*x*_)N (Do = Sc, *x* = 0.25, 0.30 or Y, *x* = 0.25) films were deposited by direct current (DC) reactive sputtering onto Ti seeding layers. The conditions under which the Ti layers were deposited are described in detail in Cohen et al.^17^ Two-inch diameter substrates were used: ⟨100⟩ cut \p-type Si wafers, resistivity 10–30 Ω·cm, University Wafers, thickness 250 ± 25 μm. The substrates were cleaned with solvents in order of increasing polarity: acetone, isopropyl alcohol, deionized water. Dilute (4 vol %) HF was then used to remove the native oxide layer as well as surface contaminants. The substrates underwent argon and oxygen plasma cleaning to remove organic contaminants in the sputtering chamber at 10 mTorr pressure with oxygen/argon volume ratio of 1:1. Pressure in the chamber was lowered to 5 mTorr with volume ratio between argon and nitrogen 1:4. Without breaking the vacuum following deposition of the Ti seeding layers, the substrates were heated in the sputtering chamber to 673 ± 10 K. 250 W power was then applied to a 3 in. diameter magnetron loaded with a 5N purity metal alloy target (Al_1–*x*_Sc_*x*_) (*x* = 0.25, 0.30), or 3N purity metal alloy target Al_0.75_Y_0.25_ (all from Abletargets, China). Reactive DC sputtering from the metallic alloy target, Al_1–*x*_Do_*x*_, was performed for 30 min in nitrogen/argon plasma. Deposition at a rate of 3.5–4 nm/min was then continued at 523 K for 8 h to achieve the desired film thickness (2 μm).

### Film Characterization

Film thickness was measured on sample cross sections imaged with a scanning electron microscope (SEM, Zeiss Sigma 500, and Zeiss Supra 55VP, 4–8 keV). SEM images were also used to estimate the mean grain size and morphology of both the surface and cross-section. Elemental analysis was performed by energy dispersive X-ray spectroscopy (EDS) using a four-quadrant detector (Bruker, FlatQUAD) installed on the Zeiss Ultra 55 SEM. Accelerating voltage was 20 kV. X-ray diffraction (XRD) patterns were collected with a TTRAX III diffractometer (Rigaku, Japan) in the Bragg–Brentano mode. Voigt profile fitting was applied to the 002 diffraction peaks (Jade, MDI). The intensity and line width of the (Al_1–*x*_Do *x*)N (Do = Sc_*x*=0.25,0.30_ or Y_*x*=0.25_), 002 diffraction peak monitored the extent of *c*-axis texture and crystallinity perpendicular to the plane of the substrate. Film in-plane stress (σ) was deduced from the change in the wafer curvature, before and after film deposition, using a DektakXT stylus profilometer. The Stoney formula was used for calculation of residual in-plane film stress.^18^, where *E* is the Young’s modulus; *h* is the thickness; ν is the Poisson’s ratio; *R* is the cantilever radius of curvature following deposition; and *R*_0_ is the initial radius of curvature. Subscripts s and f refer to the substrate and thin film, respectively. Since the doped AlN film was by far the thickest in the stack, in-plane stress was calculated neglecting the mechanical properties of other layers.

### X-ray Photoelectron Spectroscopy (XPS)

X-ray photoelectron spectroscopy (XPS, Kratos AXIS-Ultra DLD spectrometer with monochromatic Al Kα source, 15–75 W) was used for surface (8–10 nm) chemical analysis of the Al_1–*x*_Sc_*x*_N layers, *x* = 0.25, 0.3 with ScN powder as a reference sample. In an attempt to eliminate beam-induced charging artifacts, the energy scale was referenced to the theoretical binding energy of N 1s in AlN. This somewhat arbitrary choice was cross-checked using a dedicated experimental procedure in which the total surface charging can be evaluated at any given time and referred to the zero-exposure limit. Thus, consistency between samples was kept high and the evaluation of fine changes in Sc binding energies approached accuracy ±<50 meV. Ar-ion sputtering at 4 keV beam energy was used, starting with short sputtering steps (∼1 μA, on a 5 × 5 mm^2^ raster for 30 s) and increasing gradually, in order to capture fine surface details and to identify potential beam-induced effects. In general, the layers exhibited robust behavior under the stepwise sputtering such that no metallic clusters were formed. Yet, Sc-oxide dilution upon increased sputtering time should be taken into account.

### X-ray Absorption Spectroscopy

To study the oxidation state and near neighbor environment of Sc and Y dopants in thin films of Al_1–*x*_Sc_*x*_N and Al_1–*x*_Y_*x*_N, X-ray absorption spectra (XAS) were collected at beamlines 8-BM (for Sc K-edge) and 7-BM (for Y K-edge) of the National Synchrotron Light Source (NSLS-II) at Brookhaven National Laboratory, New York. Thin film data were collected in fluorescence mode, whereas transmission mode was used for the yttrium foil. ScN and yttria powders were measured in the fluorescence mode (Table 1). EXAFS analysis was performed using the Demeter package to derive quantitative structural information concerning the near neighbor environment of Sc and Y in AlScN or AlYN.

**Table 1:** Thin Film, Foil, and Powder Samples Examined by XAS

samples	edge	measurement mode
Al_0.75_Sc_0.25_N, Al_0.70_Sc_0.30_N	Sc K-edge	fluorescence
ScN powder	Sc K-edge	fluorescence
Y_2_O_3_ powder	Y K-edge	fluorescence
Al_0.75_Y_0.25_N	Y K-edge	fluorescence
Y foil	Y K-edge	transmission

## Results and Discussion

### XRD, SEM, and EDS Characterization of Thin Films of Al_1–*x*_Do_*x*_N (Do = Sc, *x* = 0.25, 0.30 or Y, *x* = 0.25)

**Figure 1:**
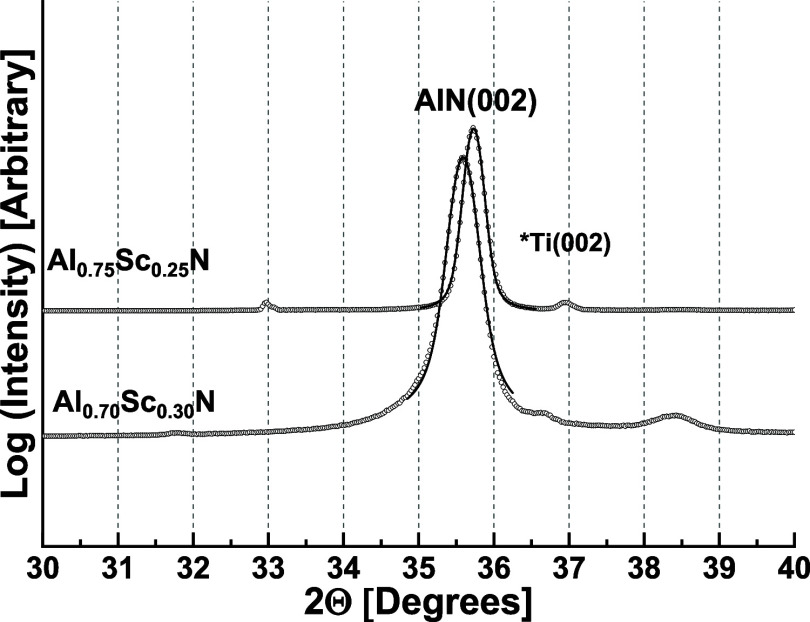
XRD patterns of Al_0.75_Sc_0.25_N and Al_0.70_Sc_0.30_N thin films grown on ⟨100⟩ cut Si wafers with a 50 nm Ti seeding layer. The substrate is tilted 3° in order to minimize diffraction from the Si substrate.

**Figure 2:**
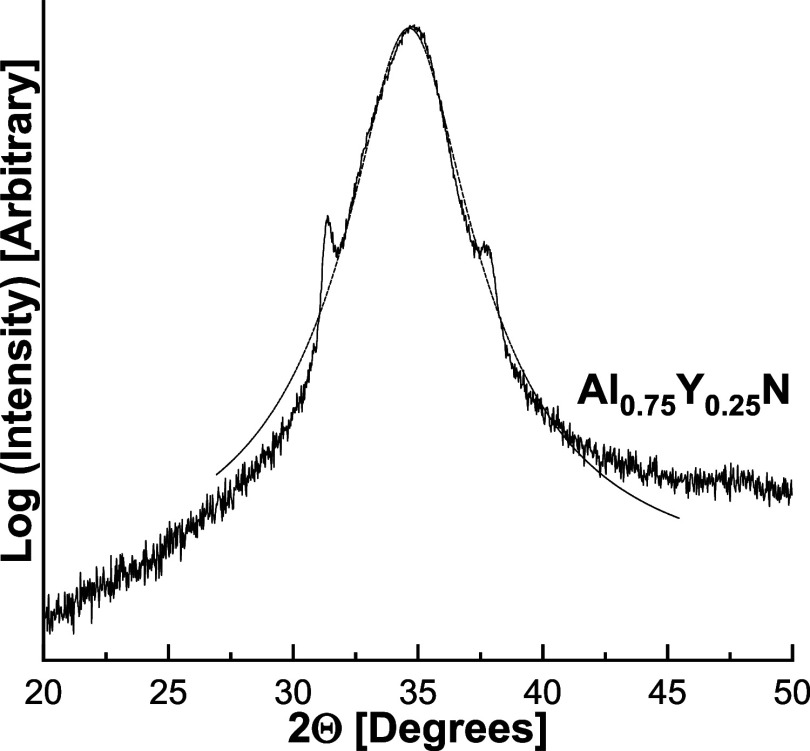
XRD pattern of an Al_0.75_Y_0.25_N thin film grown on ⟨100⟩ cut Si with a 50 nm Ti seeding layer. A pattern with full 2θ range is included in the SI (Figure S1). The substrate has been tilted 3° in order to minimize diffraction from the Si substrate.

**Figure 3:**
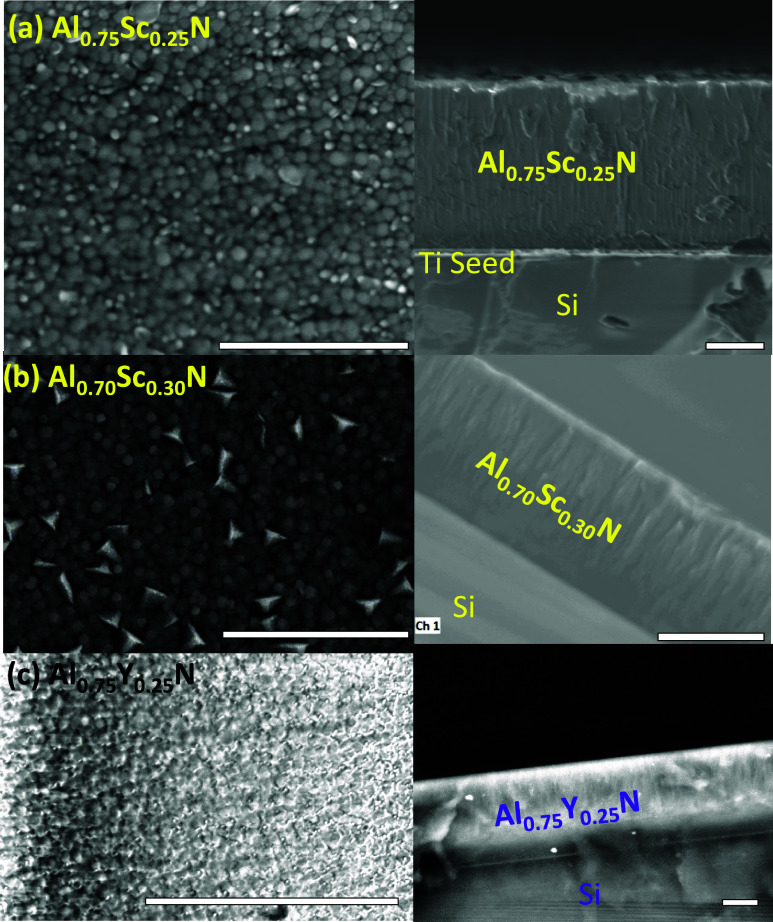
SEM images of the surface and cross-section of thin films (a diamond pen was used to prepare cross sections) of (a) Al_0.75_Sc_0.25_N, reproduced with permission from ref (17). (b) Al_0.70_Sc_0.30_N and (c) Al_0.75_Y_0.25_N, respectively. Pebble-like grains (84–94 nm) appear on the surface in panels (a) and (b), along with columnar growth. Grain size decreases with increasing Sc concentration. Disoriented, abnormal grains are observed on the surface of Al_0.70_Sc_0.30_N. We noted in our earlier report^17^ that during reactive sputtering of AlScN, the final deposition temperature influences the number of abnormally oriented grains visible in SEM images. In panel (c), individual grains and grain size on the film surface are difficult to distinguish, indicating a tendency to poor crystallinity, as has been reported in the literature.^15^ All scale bars indicate 1 μm.

XRD patterns of AlScN films were dominated by a strong wurtzite 002 diffraction peak at 2θ = 35.73° for Al_0.75_Sc_0.25_N (Figure 1) and at 2θ = 35.58° for Al_0.70_Sc_0.30_N (Figure 1). In the case of Al_0.75_Y_0.25_N, three peaks were observed at 2θ = 31.31, 34.64 and 37.7° (Figure 2 and Supporting Section S1), which could be indexed as being due to the (100), (002), (101) planes of the *P*6_3_*m* lattice, *c* = 5.11 Å, *a* = *b* = 3.29 Å, γ = 120°. The minimum crystal size perpendicular to the plane of the substrate, calculated by the Scherrer formula from the full width at half height of the 002 diffraction peak, was 57 and 38 nm for Al_0.75_Sc_0.25_ and Al_0.70_Sc_0.30_N, respectively. The comparable value was 2.5 nm for the Al_0.75_Y_0.25_N film. Scanning electron microscopy (SEM) imaging of the (AlSc)N films surface and cross-section (Figure 3a,b) shows pebble-like grains of mean transverse size of 84–92 nm, with columnar growth. In the case of Al_0.70_Sc_0.30_N, disoriented, pyramid-shaped grains occupy a minor fraction of the surface area. Although metal stoichiometry of the deposited films may differ from that of the alloy target, EDS showed negligible change, i.e., Al_0.75_Sc_0.25_, Al_0.70_Sc_0.30_, and Al_0.75_Y_0.25_ (see also Supporting Section S2). In-plane compressive stress of the films was 60 and 200 MPa for Al_0.75_Sc_0.25_N and Al_0.70_Sc_0.30_N, respectively, and 1400 MPa for Al_0.75_Y_0.25_N films, as calculated from the change in Si wafer curvature, using the Stoney formula.^18^ Transverse grain size could not be determined for Al_0.75_Y_0.25_N thin films due to excessive charging in the electron microscope.

### X-ray Absorption Fine Structure (XAFS) of Sc-Doped Aluminum Nitride

**Figure 4:**
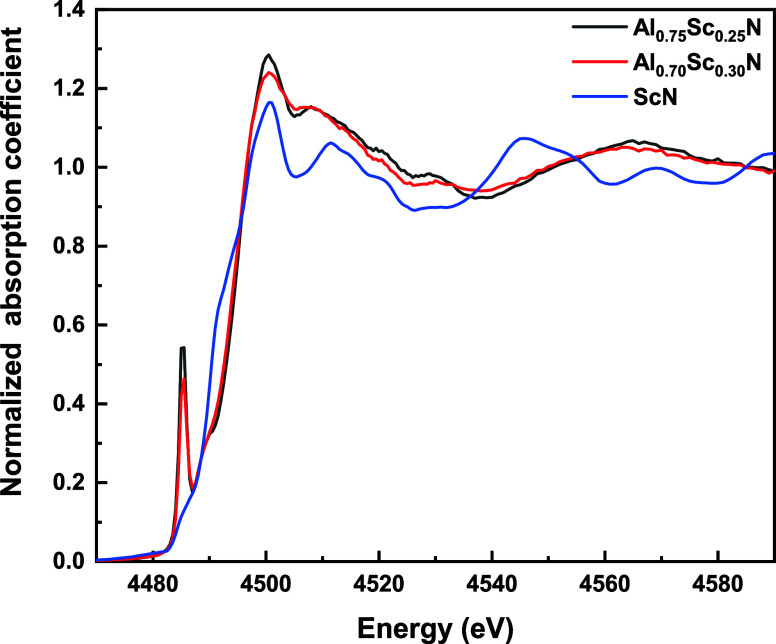
Sc K-edge XANES spectra of Al_0.75_Sc_0.25_N (black trace), Al_0.7_Sc_0.3_N (red trace) thin films, and ScN (blue trace) powder. Note the absence of a pre-edge peak for ScN.

The near neighbor environments of Sc^3+^ dopants were examined by Sc K-edge X-ray absorption spectroscopy (XAS). Both EXAFS and XANES measurements were analyzed. The presence of the pre-edge peak, denoted A, in the XANES spectra (Figure 4) of both Al_0.75_Sc_0.25_N and Al_0.70_Sc_0.30_N, points to an asymmetric environment, i.e., the Sc^3+^ ion is not located at an inversion center.^19−22^ This asymmetry would be expected for the tetrahedrally coordinated (CN = 4) scandium ion. By contrast, the pre-edge peak is absent in the XANES spectrum of ScN, which is consistent with the octahedrally coordinated environment (CN = 6) of the scandium ion in the rock-salt (*Fm*3̅*m*) lattice. The pre-edge peak is weaker for Al_0.70_Sc_0.30_N than for Al_0.75_Sc_0.25_N. Possible explanations for this change include: (i) a homogeneous model for *x* = 0.30, in which Sc ions are all located in a relatively more symmetric environment, averaged over time, than the Al ions; or (ii) assuming a heterogeneous model for *x* = 0.30, in which some (unknown) fraction of the Sc ions reside in a symmetric local environment and the remainder do not. Due to the ensemble-average nature of X-ray absorption spectroscopy,^23^ it is not possible to reliably distinguish between these two models on the basis of the XANES data alone.

**Figure 5:**
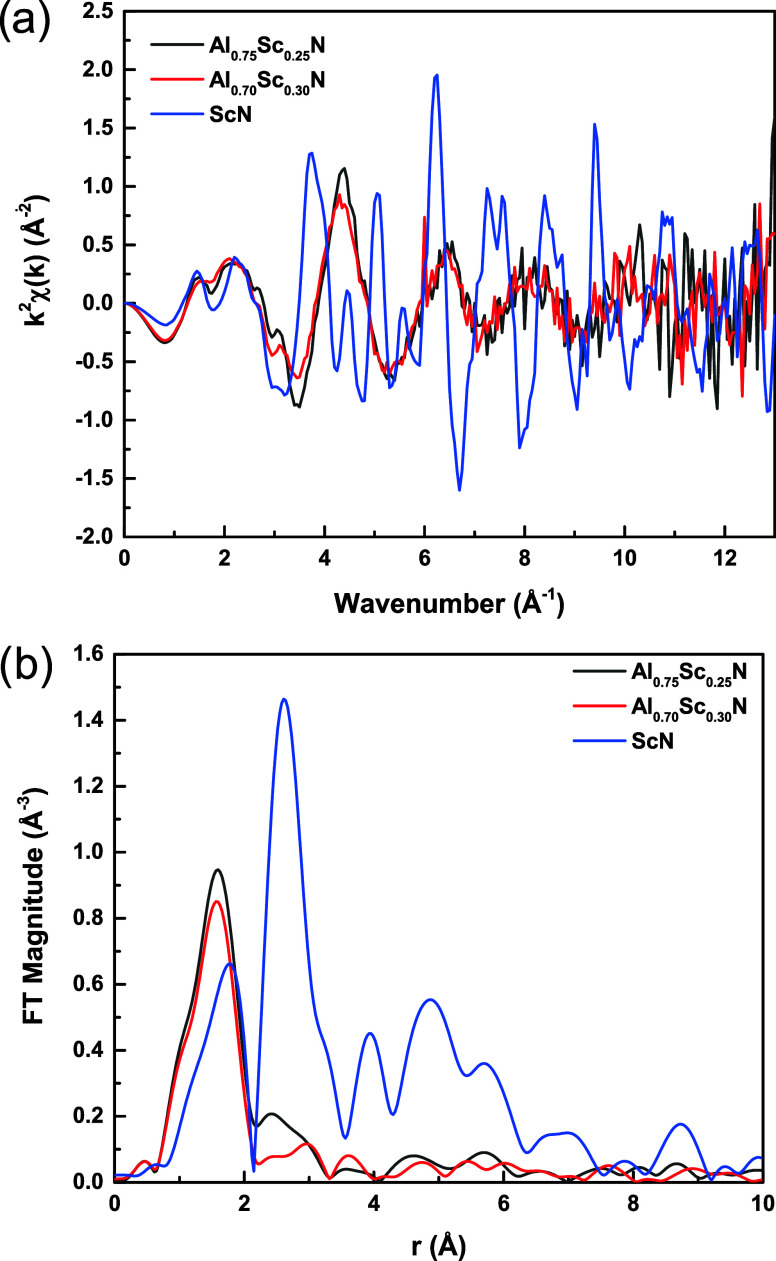
(a) *k*^2^-weighted EXAFS spectra of Al_0.75_Sc_0.25_N (black trace) and Al_0.7_Sc_0.3_N (red trace) films and ScN (blue trace) powder; (b) Fourier transform magnitude of the *k*^2^-weighted EXAFS spectra shown in panel (a). The *k*-range used for the Fourier transform was 2–9 Å^–1^.

**Figure 6:**
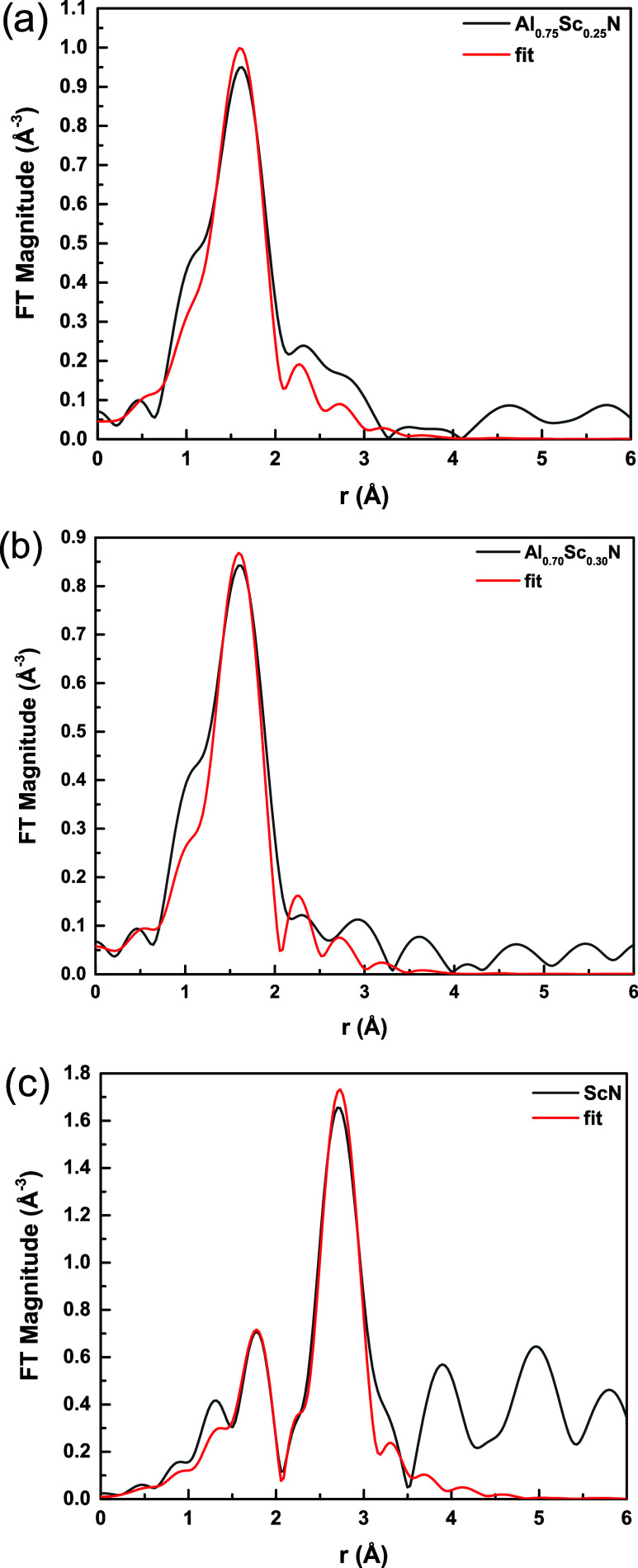
Fourier transform magnitude of *k*^2^-weighted EXAFS spectra of (a) Al_0.75_Sc_0.25_N; (b) Al_0.7_Sc_0.3_N; (c) ScN, accompanied by theoretical fits. Best fit parameters are tabulated in Table 2. The *k*-ranges used in the Fourier transform were 2.5–9.5 Å^–1^ for Al_0.75_Sc_0.25_N and Al_0.7_Sc_0.3_N and 3–11 Å^–1^ for ScN. The *r-*ranges used were 1.0–2.205, 1.0–2.607, and 1.0–3.229 Å for Al_0.75_Sc_0.25_N, Al_0.7_Sc_0.3_N, and ScN, respectively.

Examining the EXAFS spectra (Figure 5a) demonstrates that the Fourier transform (Figure 5b) magnitudes of *k*^2^-weighted EXAFS spectra for the ScN powder reveal a strong second shell peak near 2.61 Å and a weaker first shell peak in *r*-space. For Al_1–*x*_Sc_*x*_N, *x* = 0.25, 0.30, the first shell peak is prominent, and the peak position is shifted to smaller spacings relative to those observed for ScN. Models for each sample were calculated using the Demeter data analysis package with FEFF6 code fit to the EXAFS data (Figure 6a–c). As is evident in the XANES spectrum, the coordination number of Sc would be expected to be 4. Analysis of Sc K edge EXAFS could not independently verify the 4-coordinated Sc environment model due to the relatively large error bars in the coordination numbers (relative error of ca. 25%). Thus, for confirming the value of CN = 4 expected for Sc, we examined the mean value of the passive electron reduction factor, *S*_0_^2^,^24^ that was allowed to vary for each material, assuming a fixed number of nearest neighbors (4), and obtained from the fit of EXAFS theory to the data. The fitting results for Al_0.75_Sc_0.25_N, Al_0.70_Sc_0.30_N, and ScN are given in Table 2. As expected, the *S*_0_^2^ values for the thin films were found to lie between 0.7 and 1.0, thereby supporting the model we have used. However, the ScN reference spectrum measured on a powder sample exhibited strong self-absorption. As such, the amplitude factor obtained was smaller than expected (Table 2), but the interatomic distances, not perturbed by the self-absorption effect, are very informative for choosing the proper model for Sc placement in AlN.

**Table 2:** EXAFS Fitting Results for (Al_0.75_Sc_25_)N, (Al_0.70_Sc_30_)N, ScN Powder, (Al_0.75_Y_25_)N, and Y Foil^a^,^b^

sample	path	CN	*S*_0_^2^	*R* (Å)	σ^2^ (Å^2^)	Δ*E*_0_ (eV)
Al_0.75_Sc_0.25_N	Sc–N	4	0.81 ± 0.27	2.11 ± 0.03	0.005 ± 0.005	1.0 ± 3.4
Al_0.70_Sc_0.30_N	Sc–N	4	0.69 ± 0.17	2.12 ± 0.02	0.004 ± 0.004	–0.5 ± 2.6
ScN	Sc–N	6	0.34 ± 0.05	2.27 ± 0.03	0.001 ± 0.003	2.4 ± 3.9
	Sc–Sc	12	0.34 ± 0.05	3.19 ± 0.01	0.001 ± 0.001	–3.0 ± 1.5
Al_0.75_Y_0.25_N	Y–N	3.7 ± 1.3	0.82	2.24 ± 0.03	0.000 ± 0.006	–4.2 ± 3.3
Y foil	Y–Y	12	0.82 ± 0.16	3.61 ± 0.01	0.014 ± 0.002	–0.8 ± 0.9
AlN^25^	Al–N	4		1.92 ± 0.01		

a CN is the coordination number (i.e., number of nearest neighbors at distance *R* per absorbing atom); *R* is the first near neighbor distance; σ^2^ is the mean squared relative bond disorder (also referred to as the EXAFS Debye–Waller factor); Δ*E*_0_ is the correction in the photoelectron energy origin, and *S*_0_^2^ is the amplitude reduction factor.

b Reference to structural AlN data from the literature.^25^

**Figure 7:**
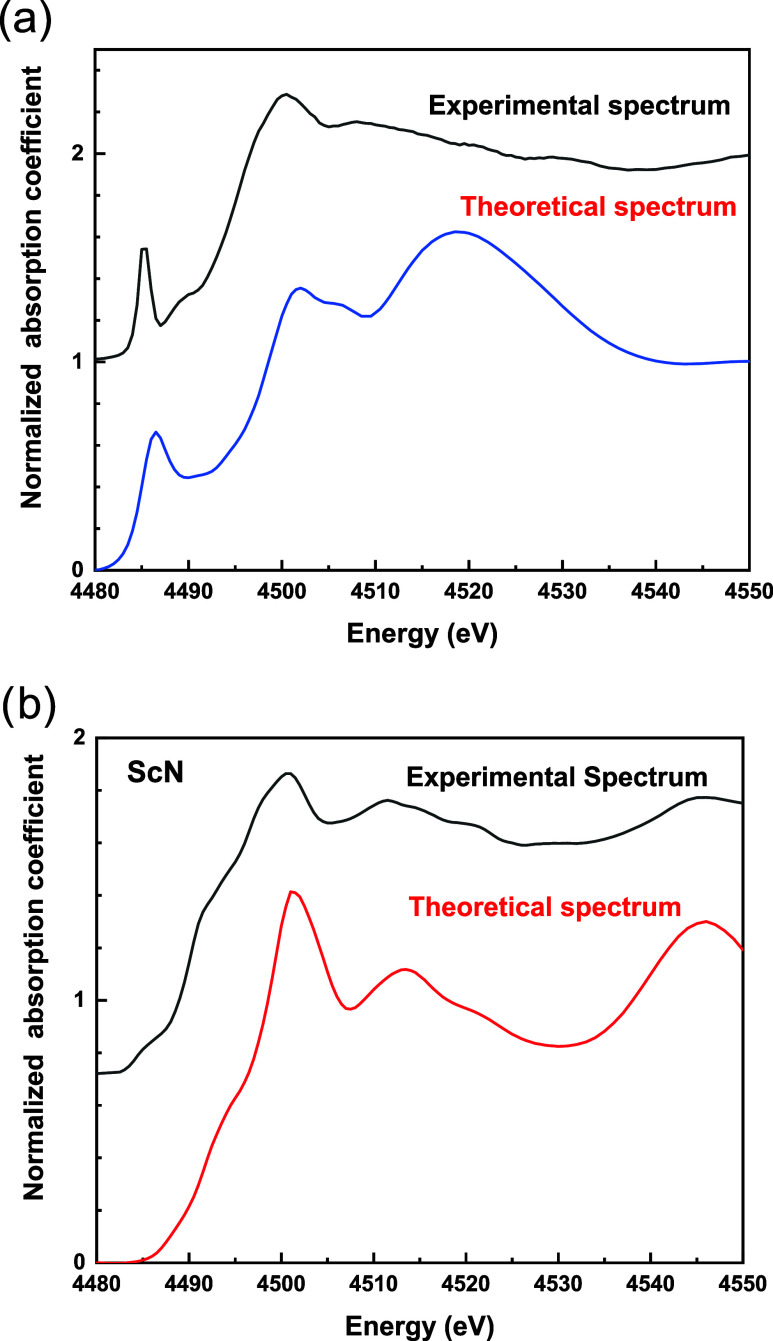
(a) Measured (black curve) and calculated (blue curve) XANES spectra of a Al_0.75_Sc_0.25_N thin film; (b) measured (black curve) and calculated (red curve) XANES spectra of ScN powder.

The results presented in Table 2 demonstrate that the first shell Sc–N distances in tetrahedrally coordinated Al_0.75_Sc_0.25_N and Al_0.70_Sc_0.30_N are similar, 2.11 ± 0.03 and 2.12 ± 0.02 Å respectively, whereas the Sc–N distance in octahedrally coordinated ScN is larger, 2.27 ± 0.03 Å. Hence, 0.16 Å shortening occurs when the scandium ion local environment changes from octahedral to tetrahedral configuration. The second shell, Sc–Sc distance in ScN, was found to be 3.19 ± 0.01 Å. By subtracting the N_CN=4_^3–^ Shannon crystal radius (1.32 Å)^9^ from the Sc–N distance in AlScN, the crystal radius of Sc_CN=4_^3+^ species of 0.79 ± 0.02 Å may be estimated. The same calculation can be made for Al_CN=4_^3+^ using the Al–N distance from the literature (1.901 Å).^25^ This results in a crystal radius for Al_CN=4_^3+^ of 0.58 Å, i.e., only 5 pm larger than the value reported by ref (9). Consequently, we may assert that the difference between the crystal radii of the solute and solvent atoms for Al_0.75_Sc_0.25_N and Al_0.70_Sc_0.30_N is ∼36%. We have performed theoretical modeling of the Sc K-edge XANES spectra using FEFF9 code,^26^ calibrating the input parameters using the agreement between the experimental and theoretical ScN spectra (Figure 7a). The same parameters were used to obtain simulated XANES spectra of a structure of Al_0.75_Sc_0.25_N (Figure 7b). The resulting simulation displays a spectrum with the same prominent pre-edge peak as observed in the experiment.

### X-ray Photoelectron Spectroscopy (XPS) of Sc-Doped Aluminum Nitride

**Figure 8:**
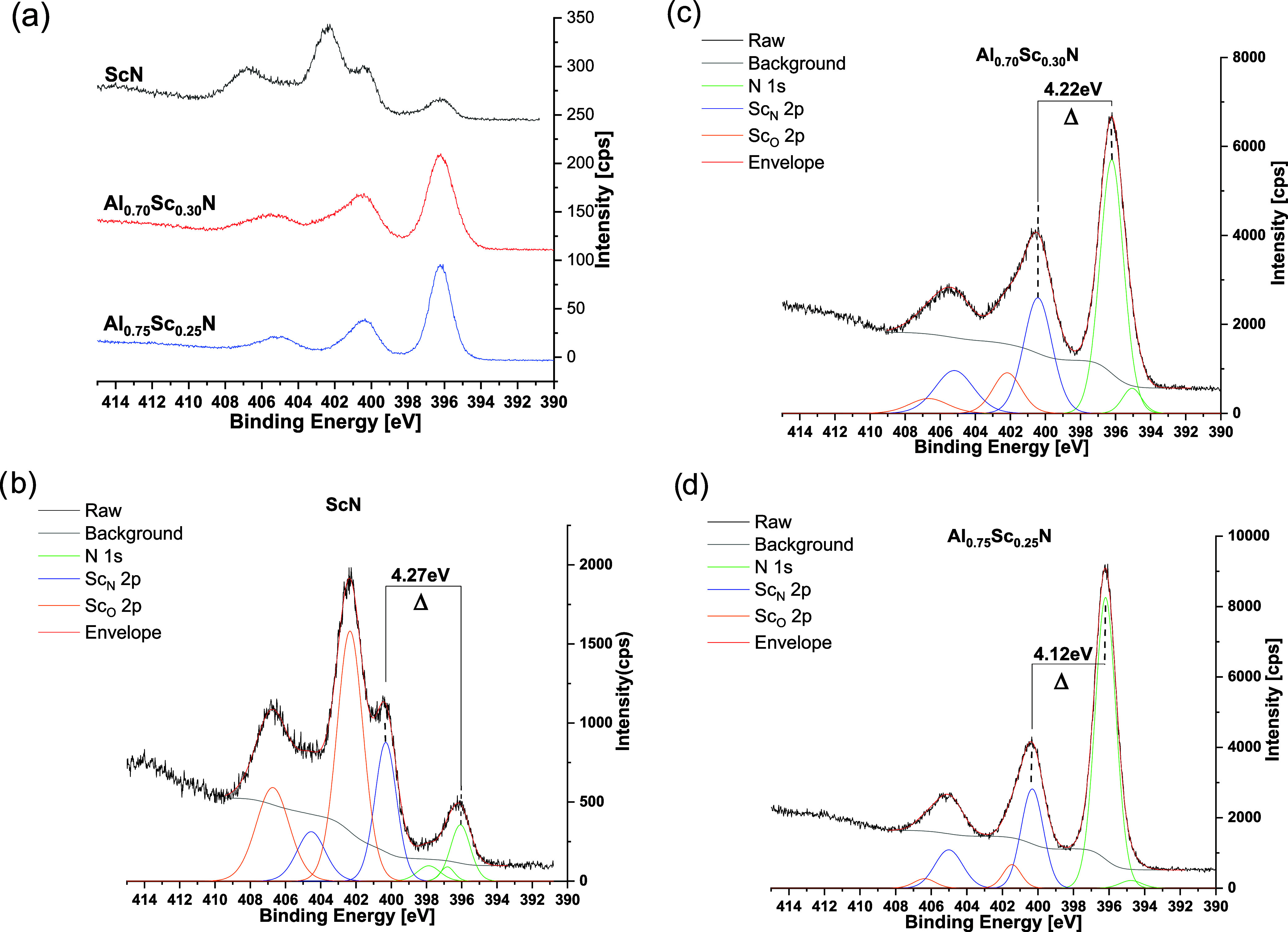
(a) X-ray photoelectron spectra of the binding energy of N 1s and Sc 2p electrons in Al_0.75_Sc_0.25_N (blue trace) and Al_0.7_Sc_0.3_N (red trace) thin films and ScN (black trace) powder samples. Profile fitting was performed for each of the three samples: (b) ScN; (c) Al_0.7_Sc_0.3_N; (d) Al_0.75_Sc_0.25_N in order to estimate the amplitude of the observed increase in Δ with increase in Sc doping in the wurtzite lattice.

Complementary information was obtained from XPS analysis. This surface-sensitive technique, typically probing to a depth ≤15 nm below the film surface, revealed significant surface oxidation, accompanied by surface Sc-depletion. Therefore, for any quantitative analysis of fine details in the Sc oxidation states, we relied on (1) comparison to a reference ScN sample and (2) stepwise Ar-ion etching that enabled gradual removal of surface species, including close inspection of beam-induced artifacts. Figure 8a presents the N 1s + Sc 2p spectral window, recorded from a reference ScN powder and from Al_1–*x*_Sc*_x_*N (*x* = 0.25 and 0.30) films after etching the surface by Ar-ions. Notably, the reference sample exhibits three nitrogen signals (green curves in Figure 8b) and two Sc doublets, attributed to ScN (blue components) as well as to Sc_2_O_3_ (orange components). Both Sc doublets and the main N 1s signal provide useful references for the ternary thin films. As shown in Figure 8c,d, the etched films consist of a single N 1s signal and two Sc doublets. Similarity to the reference spectrum in Figure 8b is apparent. Details of the XPS-derived surface stoichiometry are provided in the Supporting Information file (Section S3 and Table S4). Notably, the energy difference, Δ, between the N 1s peak and the ScN-related doublet reveals small variations for the different samples, as indicated in Figure 8. These variations propose a slightly more electron-rich environment for Sc at low-*x* values, ≈100 meV in magnitude (compare Figure 8c,d). This latter observation does, of course, suffer from the possible presence of unknown potential effects on the nitrogen signal and, therefore, must be considered only as a secondary support for the XAS result.

Two comments should be added in this respect. First, the XPS of nonetched surfaces (data not shown) are in agreement with the Sc binding energy behavior as described above. Second, for a ≈100 meV difference in Δ*v*alues between (nominally) *x* = 0.25 and 0.30 (Figure 8c,d), the actual difference may be larger: coexistence of two environments should be analyzed as two superimposed signals. Nevertheless, the XPS data are consistent with the presence of two Sc–N local environments: CN = 4, dictated by the AlN host, dominant at low Sc concentrations, and CN = 6 environment, which increases as the Sc concentration increases.

Our suggestion that at 30 mol % Sc doping in sputtered AlN thin films, a small fraction of the Sc^3+^ ions are coordinated by 6 rather than by 4 N atoms does not contradict the well-documented increase in the piezoelectric strain coefficient *d*_33_. Rather, wurtzite lattice destabilization, brought about by the attempt to incorporate a large cation that prefers coordination number (CN) = 6, can indeed explain this effect. A destabilized, mechanically softer lattice is more readily deformed in the presence of an applied stress or electric field. As reviewed by Ambacher et al.,^27^ the stiffness coefficient C_33_(*x*) decreases due to the alloying of wurtzite-AlN with rock-salt-ScN, but at the same time, the piezoelectric coefficient *d*_33_(*x*) increases very sharply up to the miscibility limit. This finding is similar to the mechanism suggested for the case of the morphotropic phase boundary (MPB) in solid solutions of PbZrO_3_–PbTiO_3_ (PZT),^28^ although, unlike (Al, Sc)N, this system displays full miscibility. In PZT, the coexistence of different structures, even if nonpiezoelectric,^29^ increases the piezoelectric response in the vicinity of the MPB because it destabilizes the lattice. So, the more compliant the lattice, the larger the response.

### X-ray Absorption Fine Structure (XAFS) of Y-Doped Aluminum Nitride

**Figure 9:**
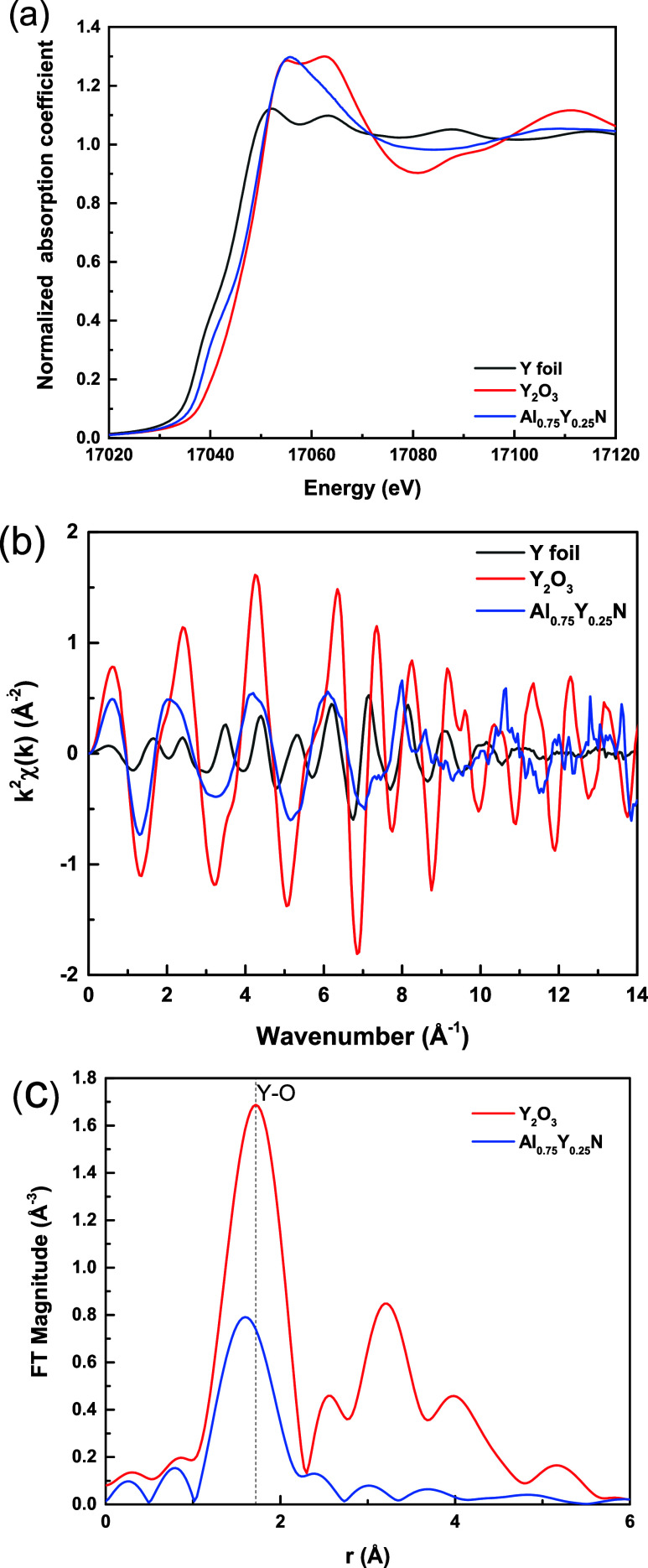
(a) Normalized Y K-edge XANES spectrum of Al_0.75_Y_0.25_N. For comparison, the spectra of Y foil and Y_2_O_3_ are included. (b) The *k*^2^-weighted EXAFS spectra of Al_0.75_Y_0.25_N thin film, Y_2_O_3_ powder, and Y foil. (c) Fourier transform magnitude of the *k*^2^ -weighted χ(*k*) spectra. The *k* range for the Fourier transformation is 2–7.5 Å^–1^.

**Figure 10:**
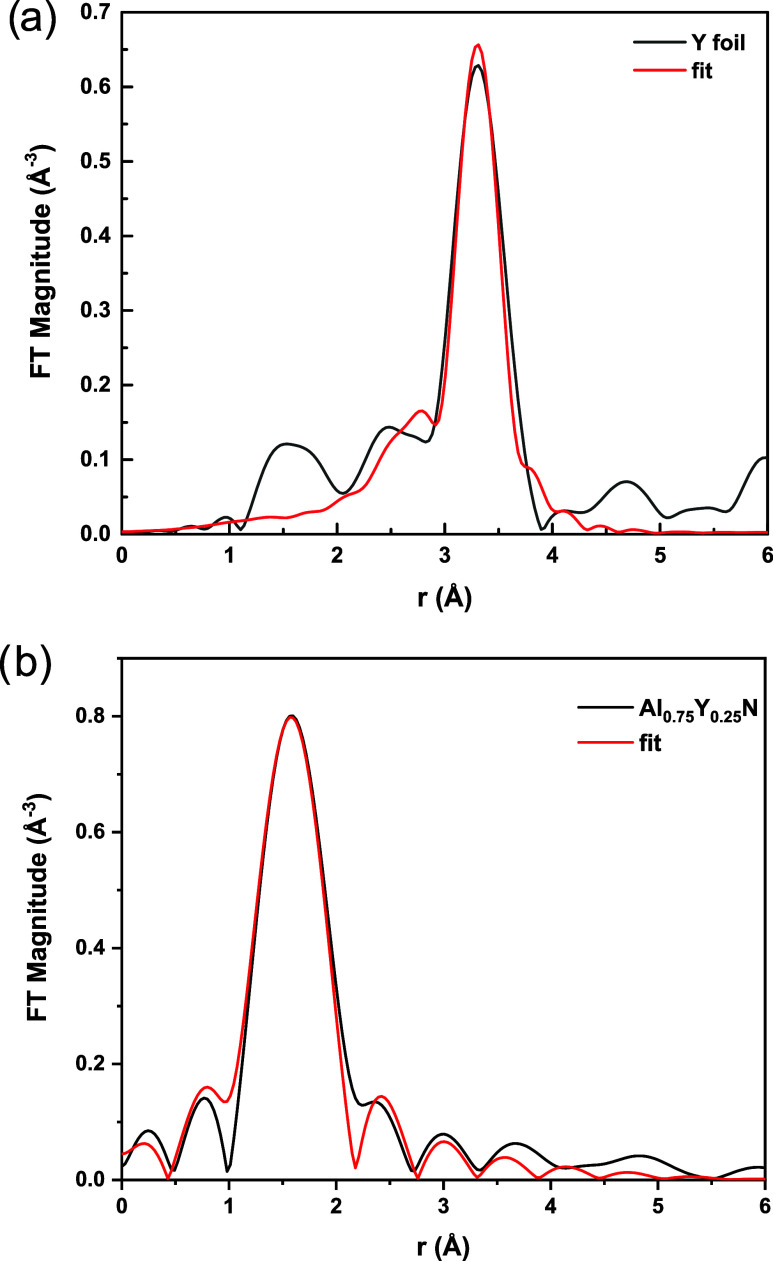
Measured (black curve) and fitted (red curve) Fourier transform magnitude of the *k*^2^-weighted EXAFS spectrum for (a) Y foil. The *k* range for the Fourier transform is 2–12 Å^–1^. The *r* range is 2.6–4 Å; (b) Al_0.75_Y_0.25_N film. The *k* range for the Fourier transform is 2–7.5 Å^–1^. The *r* range is 1.3–3.3 Å.

K-edge XANES spectra of Y foil, Y_2_O_3_ powder, and Al_0.75_Y_0.25_N thin film samples are shown in Figure 9a. Although present, the pre-edge peak, indicative of local asymmetry for Y in the thin film, is substantially weaker than that observed for the Sc-doped samples. This might be anticipated for a 4d atom, in comparison to a 3d atom, due to broadening effects.^30^ The shape of the Y K-edge XANES spectrum of Al_0.75_Y_0.25_N differs from that of double fluorite Y_2_O_3_ (Figure 9), indicating that the local environment of Y^3+^ in Al_0.75_Y_0.25_N is unlike that in Y_2_O_3_, as expected. In comparison to Y_2_O_3_, the intensity of the first shell peak obtained by FT of the *k*^2^ weighted absorption spectrum (Figure 9b) is much weaker and the position of the peak is shifted to smaller *r* values in Al_0.75_Y_0.25_N (Figure 9c). From this, we conclude that the mean coordination number in Al_0.75_Y_0.25_N is smaller than 6. Y foil measured on the same beamline was analyzed to obtain the passive electron reduction factor (S_0_^2^) of Y. The value for *S*_0_^2^ (0.82) was used in fitting the Al_0.75_Y_0.25_N spectrum (Figure 10). The coordination number (CN), which was allowed to vary for Y in Al_0.75_Y_0.25_N, is 3.7 ± 1.3. The Y–N bond length in Al_0.75_Y_0.25_N was found to be 2.24 ± 0.03 Å, while the literature value (ICSD #37413) for YN bond length in the rock-salt structure, octahedral coordination, is 2.438 Å. Accordingly, the Y_CN=4_^3+^ crystal radius is estimated to be 0.92 ± 0.01 Å compared to 1.118 Å for Y_CN=6_^3+^^9^. Consequently, the difference between the crystal radius of the solute and solvent atoms for Al_0.75_Y_0.25_N is ∼58%.

## Conclusions

In summary, the local structure and chemical environment of Sc and Y in predominantly ⟨002⟩ textured, Al_1–*x*_Do_*x*_N (Do = Sc, *x* = 0.25, 0.30 or Y, *x* = 0.25) sputtered thin films with wurtzite symmetry were investigated using XRD, XAS and XPS techniques. We present evidence from X-ray absorption spectroscopy that at the relatively low doping levels investigated, both Sc^3+^ and Y^3+^ ions substitute for Al^3+^ in the wurtzite lattice, thereby assuming a coordination number of four. On this basis, the effective size of the ion species in their respective coordination state could be calculated. Introducing dopants causes an increase in lattice strain, which is manifested macroscopically as increased in-plane compressive stress, particularly in the case of Y-doped AlN. By modeling the scandium local environment, EXAFS is able to suggest that a small fraction of the dopant ions experience an increase in coordination number from 4 to 6 when the dopant concentration is increased from 25 to 30 mol %. In other words, a small population of scandium ions appears to experience a change from tetrahedral to octahedral coordination at a dopant concentration significantly lower than that reported for the global wurtzite to rock-salt phase transition (42 mol % Sc^13,31,32^). In the ScN rock-salt lattice, Sc ions are all octahedrally coordinated and are in a centrosymmetric local environment. Our proposed heterogeneous model for the 30 mol % sample, based also on the weakening of the pre-edge peak in the XANES spectra, would therefore support some partitioning of Sc ions into rock-salt-like ScN_*x*_ clusters within the wurtzite matrix. XPS provides supporting evidence for this observation. Given that the Sc ions are responsible for an increase in piezoelectric response observed for doped AlN, it remains uncertain whether tetrahedrally coordinated or octahedrally coordinated Sc ions (or both) are responsible.

## Supporting Information Available

Section S1: expanded 2θ range of the XRD pattern for the Al_0.75_Y_0.25_N sample; Section S2: EDS analysis of the (Al,Sc)N films and (Al,Y)N film; Section S3: XPS analysis of (Al, Sc)N films (PDF)
