# Supplementary material for: Local Environment of Sc and Y Dopant Ions in Aluminum Nitride Thin Films
Source: ACS Appl Electron Mater. 2024 Jan 19;6(2):853–61. doi: 10.1021/acsaelm.3c01390 (PMC10902843; doi:10.1021/acsaelm.3c01390)
Supplement: Supplementary file 1 — el3c01390_si_001.pdf [file el3c01390_si_001.pdf]

Supporting Information

Local environment of Sc and Y dopant ions in aluminum nitride thin films

Asaf Cohen[1], Junying Li[3], Hagai Cohen[2], Ifat Kaplan-Ashiri[2], Sergey Khodorov[1], Ellen J. Wachtel[1], Igor Lubomirsky[1*], Anatoly I. Frenkel[3*] and David Ehre[1*]

[1]Department of Molecular Chemistry and Materials Science, Weizmann Institute of Science, Rehovot, 7610001, Israel

[2]Department of Chemical Research Support, Weizmann Institute of Science, Rehovot 7610001, Israel

[3]Department of Materials Science and Chemical Engineering, Stony Brook University, Stony Brook, NY 11794, USA

Corresponding Authors

David Ehre, Department of Molecular Chemistry and Materials Science

Weizmann Institute of Science, Rehovot 76100, Israel;

orcid.org/0000-0002-2359-2059;

Email: David.Ehre@weizmann.ac.il

Anatoly Frenkel, Department of Materials Science and Chemical Engineering

, Stony Brook University, Stony Brook, NY 11794, USA

orcid.org/0000-0002-5451-1207

Email: anatoly.frenkel@stonybrook.edu

Section S1. X-ray diffraction of Y-doped AlN.

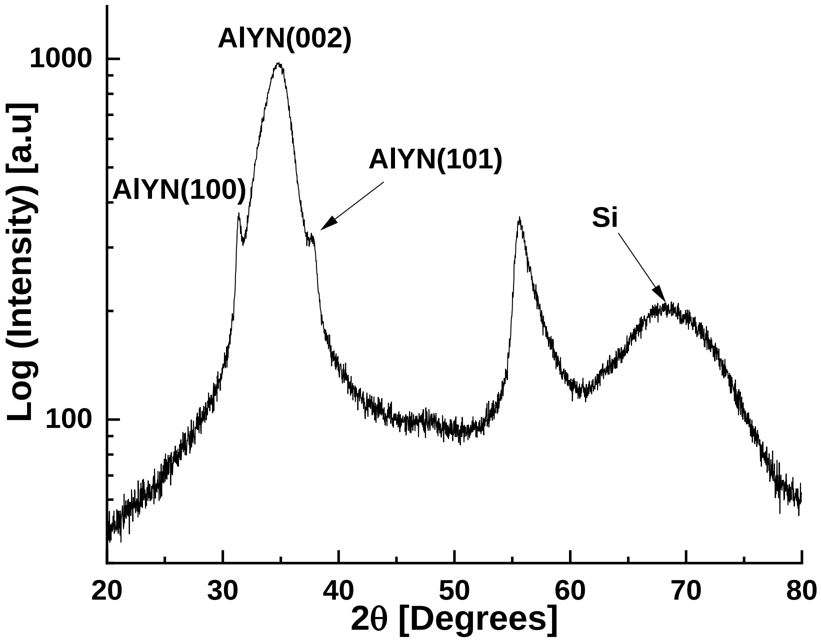

Figure S1. Expanded 2Θ range of the XRD pattern for the $Al_{0.75}Y_{0.25}N$ thin film deposited on a Si substrate. The source of the peak at approximately 2Θ = 57 degrees has not been identified. The substrate has been tilted 3 degrees from the horizontal in order to suppress the strong diffraction from the Si crystal, resulting in the broad hump at approximately 2Θ = 70 degrees.

Section S2. EDS analysis of the (Al, Sc)N films and (Al, Y)N film

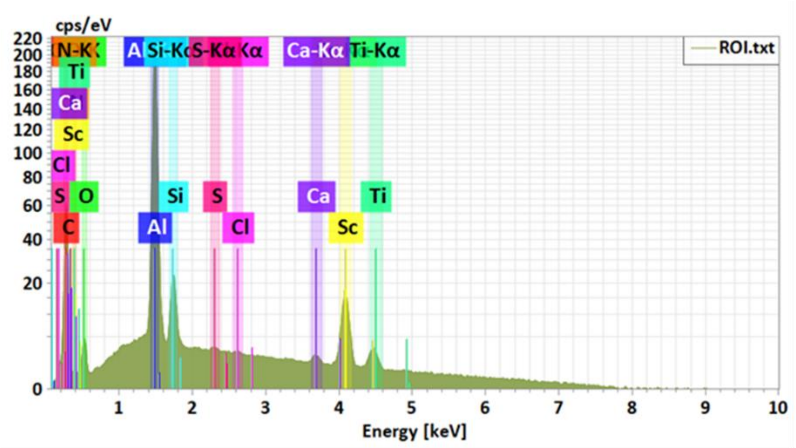

Figure S2. EDS spectrum of an (Al, Sc)N film reactively sputtered on Si/Ti from a single $Al_{0.70}Sc_{0.30}$ alloy target and measured as described in the Experimental Section .

Table S1. EDS elemental analysis of (Al, Sc)N film stoichiometry, reactively sputtered from a single $Al_{0.75}Sc_{0.25}$ alloy target[1].

Element	Atomic No.	Mass [%]	Norm.	Atom [%]	Abs.error [%]
**Aluminum**	**13**	**63.53**		**74.4**	**1.9**
**Scandium**	**21**	**36.47**		**25.6**	**1.1**

Table S2. EDS elemental analysis of (Al,Sc)N film stoichiometry, reactively sputtered from a single $Al_{0.70}Sc_{0.30}$ alloy target.

1

Element	At. No.	Netto	Mass [%]	Mass Norm. [%]	Atom [%]	abs. error [%] (1 sigma)	rel. error [%] (1 sigma)
Carbon	6	59664	0.00	0.00	0.00	0.00	0.00
Oxygen	8	4029	1.91	3.17	6.15	0.34	17.61
Aluminium	13	345395	30.28	50.30	57.98	1.37	4.54
Silicon	14	36559	5.21	8.65	9.58	0.24	4.64
Calcium	20	2014	0.64	1.07	0.83	0.06	8.68
Scandium	21	41705	22.17	36.82	25.47	0.92	4.15
		Sum	60.21	100.00	100.00		

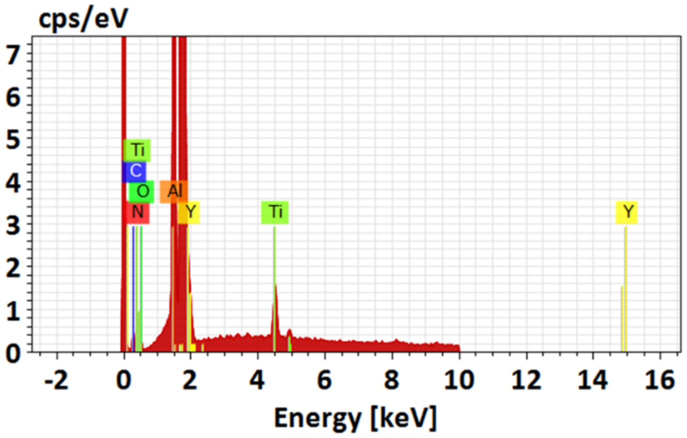

Figure S3. EDS spectrum of an (Al,Y)N film reactively sputtered on Si/Ti from a single $Al_{0.75}Y_{0.25}$ alloy target and measured as described in the Experimental Section .

Table S3. EDS elemental analysis of (Al,Y)N film stoichiometry, reactively sputtered from a single $Al_{0.70}Sc_{0.30}$ alloy target.

Acquisition 61

Element	At. No.	Netto	Mass [%]	Mass Norm. [%]	Atom [%]	abs. error [%] (1 sigma)
Carbon	6	657	0.00	0.00	0.00	0.00
Nitrogen	7	128	0.00	0.00	0.00	0.00
Oxygen	8	92	0.00	0.00	0.00	0.00
Aluminium	13	19141	49.88	49.88	76.63	1.95
Titanium	22	2896	0.00	0.00	0.00	0.00
Yttrium	39	175384	50.12	50.12	23.37	2.21
		Sum	100.00	100.00	100.00	

Section S3. XPS analysis of (Al, Sc)N film stoichiometry

Selected details of the surface composition of two Sc-containing film samples - $Al_{1-x}Sc_xN$, where x=0.25 or x=0.30 - are provided in **Table S4**: the atomic concentration ratios of (1) N/(Al+Sc); and (2) Sc/(Al+Sc). Ratios are given for 'as-received' and 'Ar-ion etched' films. **C**onsiderable deviation from the expected stoichiometry is observed for the bare surfaces, **(Table S4)**, reflecting significant oxidation as well as surface depletion of Sc. After etching with an Ar-ion beam, the expected 1:1 stoichiometry of non-oxidized N:(Al+Sc) components is nearly recovered, however Sc-depletion is still apparent, yielding x=0.15 and x=0.20 instead of 0.25 and 0.30, respectively. Therefore, the discussion in the main text, focused on binding energy differences, Δ, between the N 1s peak and the ScN-related doublet, deals with Sc concentrations even lower than those accounted for by XAS. On the other hand, the chemical shift of Sc-oxide components is sufficiently large (≥2 eV) to prevent interference of foreign signals with those of interest, i.e., the Sc-N signals. Consequently, the XPS data do provide valid secondary support for the coexistence of two local Sc-environments, which indeed change upon increased Sc concentration.

Table S4: Representative atomic concentration ratios as determined by XPS for $Al_{1-x}Sc_xN$, where x=0.25 or x=0.30, thin film samples, before and after etching the surface with an Ar ion beam. The relative experimental uncertainty is ≤ 8%.

	As-received		After surface etching	
	N/(Al+Sc)	Sc/(Al+Sc)	N/(Al+Sc)	Sc/(Al+Sc)
X=0.30	0.35	0.166	0.79	0.21
X=0.25	0.42	0.095	0.93	0.15

References:

(1) Cohen, A.; Cohen, H.; Cohen, S. R.; Khodorov, S.; Feldman, Y.; Kossoy, A.; Kaplan-Ashiri, I.; Frenkel, A.; Wachtel, E.; Lubomirsky, I. C-Axis Textured, 2–3 µm Thick $Al_{0.75}Sc_{0.25}N$ Films Grown on Chemically Formed TiN/Ti Seeding Layers for MEMS Applications. *Sensors-Basel* 2022, *22* (18), 7041.
